# Synthesis of Lipid Mediators during UVB-Induced Inflammatory Hyperalgesia in Rats and Mice

**DOI:** 10.1371/journal.pone.0081228

**Published:** 2013-12-09

**Authors:** Marco Sisignano, Carlo Angioni, Nerea Ferreiros, Claus-Dieter Schuh, Jing Suo, Yannick Schreiber, John M. Dawes, Ana Antunes-Martins, David L. H. Bennett, Stephen B. McMahon, Gerd Geisslinger, Klaus Scholich

**Affiliations:** 1 Institute of Clinical Pharmacology, pharmazentrum Frankfurt/ZAFES, University Hospital of the Goethe-University, Frankfurt am Main, Germany; 2 Nuffield Department of Clinical Neurosciences, John Radcliffe Hospital, University of Oxford, Oxford, United Kingdom; 3 Wolfson CARD, King’s College London, Guy’s Campus, London, United Kindgom; St. Joseph’s Hospital and Medical Center, United States of America

## Abstract

Peripheral sensitization during inflammatory pain is mediated by a variety of endogenous proalgesic mediators including a number of oxidized lipids, some of which serve endogenous modulators of sensory TRP-channels. These lipids are eicosanoids of the arachidonic acid and linoleic acid pathway, as well as lysophophatidic acids (LPAs). However, their regulation pattern during inflammatory pain and their contribution to peripheral sensitization is still unclear. Here, we used the UVB-model for inflammatory pain to investigate alterations of lipid concentrations at the site of inflammation, the dorsal root ganglia (DRGs) as well as the spinal dorsal horn and quantified 21 lipid species from five different lipid families at the peak of inflammation 48 hours post irradiation. We found that known proinflammatory lipids as well as lipids with unknown roles in inflammatory pain to be strongly increased in the skin, whereas surprisingly little changes of lipid levels were seen in DRGs or the dorsal horn. Importantly, although there are profound differences between the number of cytochrome (CYP) genes between mice and rats, CYP-derived lipids were regulated similarly in both species. Since TRPV1 agonists such as LPA 18∶1, 9- and 13-HODE, 5- and 12-HETE were elevated in the skin, they may contribute to thermal hyperalgesia and mechanical allodynia during UVB-induced inflammatory pain. These results may explain why some studies show relatively weak analgesic effects of cyclooxygenase inhibitors in UVB-induced skin inflammation, as they do not inhibit synthesis of other proalgesic lipids such as LPA 18∶1, 9-and 13-HODE and HETEs.

## Introduction

Inflammatory hyperalgesia is mediated through sensory changes in the inflamed tissue. These include mechanisms of peripheral sensitization of sensory neurons via release of proalgesic mediators by immune cells at the site of inflammation. Proalgesic components comprise of cytokines, growth factors such as the nerve growth factor NGF, reactive molecules such as nitric oxide (NO) and reactive oxygen species (ROS) as well as oxidized lipids [1], [2]. Most of these substances specifically bind to receptors at the cell membrane of sensory neurons which activate signaling cascades, leading to the activation of protein kinases PKA and PKC [3], [4]. The activated protein kinases then phosphorylate ligand gated ion channels, such as the transient receptor potential (TRP)-channels or sodium channels, thereby reducing their activation threshold [5], [6].

Apart from already known components that contribute to inflammatory hyperalgesia, many oxidized lipids have recently been identified to either directly activate or sensitize nociceptors by either interacting with transducer ion channels of the transient receptor potential (TRP)-family or sodium channels, or by recruiting immune cells to the site of inflammation. Among these lipids, eicosanoids, oxidized linoleic acid metabolites (OLAMs) and lysophophatidic acids (LPA) can be found. LPAs can be generated by the secretory phospholipase D autotaxin through cleavage of the choline group from lysophosphatidyl choline (LPC) species [7]. Recently, LPA 18∶1, the OLAMs 9-, and 13-HODE as well as the lipoxygenase product 20-HETE were identified as endogenous activators of the vanilloid receptor TRPV1 [8], [9], [10]. Moreover, the epoxylipid and 12-lipoxygenase metabolite Hepoxilin A3 (HXA_3_) has recently been identified as endogenous activators of TRPV1 and TRPA1 and inflammatory pain [11]. Additionally, leukotriene B4 acts as a chemoattractant for invading immune cells during inflammation [12]. Both activation of sensory TRP-channels and recruitment of immune cells contribute to peripheral sensitization and inflammatory hyperalgesia and enhanced pain perception [13], [14], [15].

The aim of this study was to investigate the extent to which lipid mediators are regulated in the skin and in the downstream located nociceptive systems, the DRGs and the spinal cord, during UVB-induced inflammation and if their production and regulation can explain the weak analgesic effects of cyclooxygenase inhibitors during UVB-induced skin inflammation [16], [17], [18], [19]. Therefore, the levels of prostanoids, leukotrienes, hydroxyeicosatetraenoic acids (HETEs), expoxylipids, HODEs and lysophophatidic acids were determined during peripheral UVB-induced inflammatory hyperalgesia at the time with the strongest nociceptive response. The well described model of UVB induced skin inflammation [20], [21], [22] is thought to have a high translational potential [22]. Moreover, since the number of genes for CYP enzymes differ strongly between mice on one side and rats and humans on the other side [23] we put a special focus on the comparison of levels of CYP-derived lipids.

## Materials and Methods

### Ethics Statement

All animal experiments were performed according to the recommendations in the Guide for the Care and Use of Laboratory Animals of the National Institutes of Health and approved by the local Ethics Committees for Animal Research (Darmstadt) with the permit number F95/42. The radiation procedure was performed under ketamine/xylazin anesthesia, and all efforts were made to minimize suffering.

### Animals and UVB-irradiation

For the irradiation procedure, male C57BL/6 N mice or male Sprague Dawley rats were purchased from Janvier (Le Geneset-Saint-Isle, FR) at an age of 6–8 weeks (mice) or 250–300 g (rats) and anesthesized by intraperitoneal injection of a mixture, containing ketamine (100 mg·kg^−1^) and xylazin (10 mg·kg^−1^). Hair was removed by shaving (Aeskulap® Isis GT420) and additional treatment with a commercial available hair removal creme (Pilca®) for five minutes. The radiation setup and calibration device as well as the irradiation procedure were the same as described by Bishop *et al.,* [16] except using doses of both 1000 mJ/cm^2^ and of 1500 mJ/cm^2^ for the irradiation of mice and 1000 mJ/cm^2^ for Sprague Dawley rats. During the procedure the eyes of the animals were kept moist using an ointment (Bepanthen). Shaving and hair removal were also performed in non-irradiated control animals.

### Behavioral Testing

To assess mechanical allodynia, mice were put in test cages on an elevated grid at least 1 h prior to the measurement to allow accommodation. Mechanical thresholds of the hind paws were measured using a Dynamic Plantar Aesthesiometer (Ugo Basile, Comerio, IT). A steel rod is pushed against the plantar side of the hindpaw with linear ascending force (0–5 g over 10 s, in 0.5 g/s intervals) until a fast withdrawal response was observed. Paw withdrawal latencies were determined in seconds and calculated to units of Newton (5 g = 0.049 N) and the irradiated and untreated paws were measured alternately in intervals of 5 minutes.

### Determination of Lipids from Tissue Samples by LC-MS/MS

After dissection, tissue samples were weighted. The weight ranged from 1 mg (L4–L6-DRGs) to 16 mg (skin) for murine tissue and from 3 mg (L4–L6-DRGs) to 105 mg (skin) for rat tissue. Ipsilateral DRGs of the sections L4–L6 out of one animal were pooled. The procedure was repeated for contralateral L4–L6-DRGs. Prior to the lipid extraction, tissue samples were homogenized with 5 zirconium oxide grinding balls for 3 min at 30 s^−1^ (MM400, Retsch, Haan, Germany).

#### Lipid extraction and standards

Stock solutions with 2500 ng/ml of the analytes: 5,6 EET, 8,9 EET, 11,12 EET, 14,15 EET, LTB_4_, 5-S-HETE, 12-S-HETE and 15-S-HETE and the internal standards: 5,6 EET-d11, 8,9 EET-d11, 11,12 EET-d8, 14,15 EET-d8, LTB_4_-d4, 5-S-HETE-d4, 12-S-HETE-d4 and 15-S-HETE-d4 were prepared in methanol. Working standards were obtained by further dilution with a concentration range of 0.1–250 ng/ml for all analytes. For LPAs, Stock solutions with 100,000 ng/ml of all analytes (LPA 16∶0, LPA 18∶0, LPA 18∶1, LPA 18∶2, LPA 18∶3 and LPA 20∶4) and the internal standard (LPA 17∶0) were prepared in methanol. Working standards were obtained by further dilution with a concentration range of 0.5–2500 ng/ml for all the analytes. For prostanoids, Stock solutions with 50,000 ng/ml of all analytes (PGE_2_, PGD_2_, 6-keto-PGF_1α_, TXB_2_ and PGF_2α_) and the internal standards (PGE_2_-d4, PGD_2_-d4, 6-keto-PGF_1α_-d4, TXB_2_-d4 and PGF_2α_-d4) were prepared in methanol. Working standards were obtained by further dilution with a concentration range of 0.1–1,250 ng/ml for PGE_2_, PGD_2_, 6-keto-PGF_1α_ and TXB_2_ and 0.4–5,000 ng/ml for PGF_2α_.

Sample pretreatment was performed using liquid–liquid extraction. Therefore, homogenated tissue was extracted twice with 600 µl of ethyl acetate (EETs, leukotrienes and prostanoids) or 500 µl of 1-butanol saturated with water (LPAs). The combined organic phases were removed at a temperature of 45°C under a gentle stream of nitrogen. The residues were reconstituted with 50 µl of methanol/water/butylated hydroxytoluene (BHT) (50∶50∶10^−3^, v/v/v) (EETs and leukotrienes), 50 µl of methanol (LPAs) or 50 µl of acetonitrile/water/formic acid (20∶80∶0.0025, v/v/v) (prostanoids) and then centrifuged for 2 min at 10,000 *g*, and transferred to glass vials waiting for analysis.

#### Instrumentation for lipid measurement

The LC-MS/MS system consisted of a QTrap 5500 (AB Sciex, Darmstadt, Germany) equipped with a Turbo-V source operating in negative electrospray ionization mode, an Agilent 1200 binary HPLC pump and degasser (Agilent, Waldbronn, Germany), and an HTC Pal autosampler (CTC analytics, Zwingen, Switzerland). High-purity nitrogen for the mass spectrometer was produced by a NGM 22-LC-MS nitrogen generator (cmc Instruments, Eschborn, Germany).

For the chromatographic separation of EETs and leukotrienes, a Gemini NX C18 column and precolumn were used (150×2 mm inner diameter, 5 µm particle size, and 110 Å pore size; Phenomenex, Aschaffenburg, Germany). A linear gradient was used at a flow rate of 0.5 ml/min with a total run time of 17.5 min. Mobile phase A consist of water:ammonia (100∶0.05, v/v), and mobile phase B of acetonitrile:ammonia (100∶0.05, v/v). The gradient changed from 85% A to 10% within 12 min. These conditions were held for 1 min. Then, the mobile phase shifted back to 85% A within 0.5 min and it was maintained for 4 min to reequilibrate the column.

The chromatographic separation of LPAs was achieved using a Luna C18 column (20×2 mm inner diameter, 3 µm) and a precolumn of same material, (Phenomenex, Aschaffenburg, Germany) and a linear gradient at a flow rate of 0.4 ml/min were used. Total chromatographic time was 7 min. Mobile phase A consisted of 50 mM ammonium acetate/formic acid (100∶0.2, v/v), and mobile phase B of acetonitrile/formic acid (100∶0.2, v/v). The gradient started with 60% A changing to 5% within 1 min and maintained for 2.5 min. Within 0.5 min, the mobile phase shifted back to 60% A and was held for 3 min to re-equilibrate the column.

For the chromatographic separation of prostanoids, a Synergi 4 u Hydro-RP column (150×2 mm inner diameter, 4 µm, Phenomenex, Aschaffenburg, Germany) and a precolumn of same material were used. Chromatographic separation was carried out in gradient elution mode at a flow rate of 0.3 ml/min. Total run time was 16 min. Mobile phase A consisted of water/formic acid (100∶0.0025, v/v), and mobile phase B of acetonitrile/formic acid (100∶0.0025, v/v). The linear gradient started with 90% A for 1 min and then changed to 60% A within 1 min. It was held for 1 min at 60% in phase A. Within 1 min, the mobile phase shifted to 50% in phase A and was held for 2 min. Within 2 min, the mobile phase shifted to 10% A and was held for 1 min. Composition of the gradient shifted back to 90% A in one min and it was maintained for 6 min to re-equilibrate the column.

20 µl (EETs, leukotrienes, and LPAs) or 45 µl (prostanoids) of the extracted samples were injected into the LC-MS/MS system. Quantification was performed with Analyst software version 1.5 (Applied Biosystems) using the internal standard method (isotope-dilution mass spectrometry). Ratios of analyte peak area and internal standard area (y-axis) were plotted against concentration (x-axis), and calibration curves were calculated by least-squares regression with 1/square concentration weighting.

### Data Analysis and Statistics

All data are presented as mean ± SEM. To determine statistically significant differences in all behavioral experiments, ANOVA for repeated measures was used, followed by Bonferroni’s *post hoc* correction using GraphPad Prism. For lipid measurements comparing only two groups, Student’s *t-*test was carried out. A confidence interval of 95% and a corresponding *p-*value of <0.05 were considered statistically significant.

## Results

**Figure 1:**
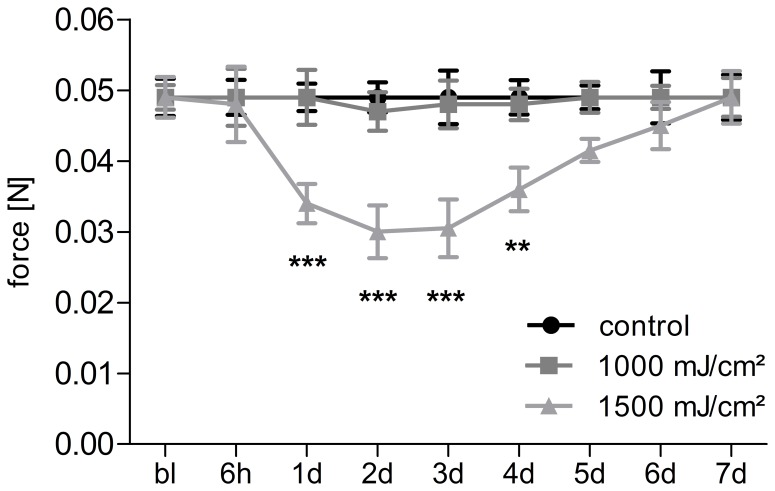
Mechanical allodynia after UV-B-irradiation in BL/6N-mice. Mice were irradiated at the plantar side of the hind paw with the indicated UV-B-doses. Mechanical thresholds were measured up to seven days post irradiation with the dynamic plantar test; bl: baseline. Data represents the average ± SEM from 10 animals per group; **p<0.01, ***p<0.001, two way repeated-measures ANOVA with Bonferroni post-hoc test.

To investigate alterations in concentrations of lipid levels during inflammatory hyperalgesia, we chose a UVB model of skin inflammation first described for rats by Bishop *et al.* [16]. First we tested two irradiation doses for BL/6 mice (1000 mJ/cm^2^ and 1500 mJ/cm^2^) because of the stronger pigmentation in mice as described previously [24]. Mice were irradiated on the plantar site of the left hind paw, while the right hind paw was not irradiated and was used as contralateral control. To evaluate mechanical hyperalgesia in mice, the paw withdrawal latency was monitored 6 h –7 d after irradiation. We observed significantly decreased mechanical thresholds of the treated mice 24 h after irradiation with a dose of 1500 mJ/cm^2^ but not with the lower dose of 1000 mJ/cm^2^. After three days the mice seemed to recover as the mechanical thresholds slowly increased and reached baseline level at day seven post irradiation (Fig. 1). Notably, the strongest and most stable decrease of mechanical thresholds is within 48 h post irradiation consistent with behavioral data from rats [16]. Therefore, at this time point skin, L4–L6-DRGs and the corresponding section of the ipsilateral dorsal horn were dissected and lipid concentrations were determined by LC-MS/MS. In mice LPAs, epoxylipids and metabolites, leukotrienes and prostanoids were measured in skin tissue, L4–L6-DRGs and the corresponding section of the dorsal horn.

**Figure 2:**
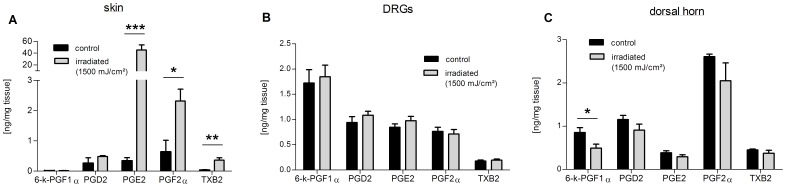
Prostanoid concentrations in skin, DRG and spinal dorsal horn samples from irradiated mice 48 h post irradiation. Concentrations of prostanoids from isolated skin (A), L4–L6-DRGs (B) and spinal dorsal horn tissue (C) of mice comparing untreated (black bars) versus irradiated skin (grey bars); 6-k-PGF1α: 6-keto-Prostaglandin F1α. Data represent mean ± SEM from six mice per group; *p<0.05, **p<0.01, ***p<0.001, student’s t-test.

Since COX-metabolites are reliable markers for inflammatory responses and contribute to peripheral inflammatory hyperalgesia [1], we first quantified the concentrations of prostaglandin (PG) D_2_, PGE_2_, PGF_2_α as well as thromboxane B_2_ (TXB_2_) and 6-Keto-PGF_1α_, the stable metabolites of TXA_2_ and PGI_2_ respectively, in the tissue samples. Consistent with previous findings from human skin [25], the concentrations of PGE_2_, were strongly elevated in murine skin. Moreover the concentrations of TXB_2_ and PGF_2_α were significantly increased at the site of irradiation in skin samples from irradiated mice (Fig. 2A). Surprisingly, none of the prostanoids increased in the lumbar DRGs L4–L6 (Fig. 2B) or in the corresponding dorsal horn sections. In the spinal tissue, 6-Keto-PGF_1α_ levels even decreased in irradiated mice as compared to untreated mice (Fig. 2C). The concentration changes of prostanoids in rat tissue were very similar to the murine tissue (Figure S1).

**Figure 3:**
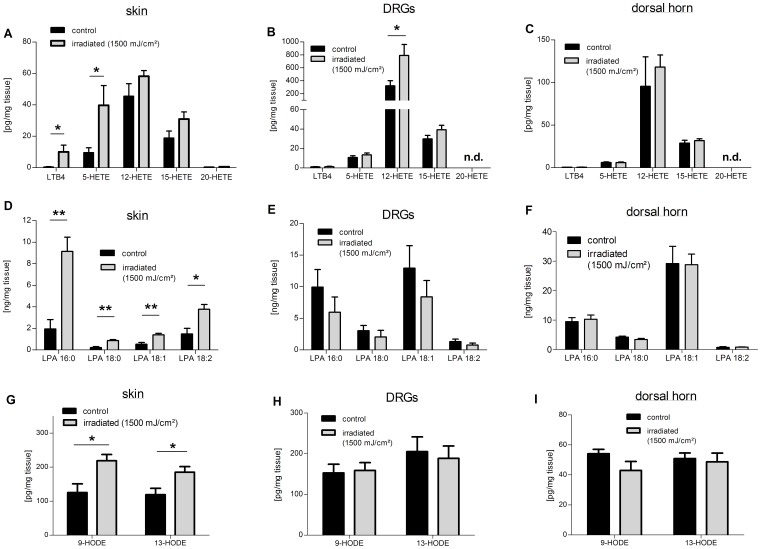
Concentrations of HETEs, LPAs and HODEs in skin, L4–L6-DRGs and spinal dorsal horn samples from irradiated mice. Shown are the concentrations of LTB_4_, 5-, 12-, 15- and 20-HETE from isolated skin (A), L4–L6-DRGs (B) and the corresponding section of the dorsal horn (C)., n.d, not detectable. (D–F) Levels of LPAs 16∶0, 18∶0, 18∶1 and 18∶2 in skin (D), L4–L6-DRGs (E) and the spinal dorsal horn (F) of irradiated versus untreated mice. (G–I) Shown are the concentrations of 9- and 13-HODE from skin (G), L4–L6-DRGs (H) and the dorsal horn of the spinal cord (I). Data represent mean ± SEM from six mice per group, *p<0.05, **p<0.01, student’s t-test.

In the group of LOX-metabolites, LTB_4_, 5-, 12-, 15- and 20-HETE were measured. Interestingly, the concentrations of two 5-LOX metabolites LTB_4_ and 5-HETE were increased in the skin of irradiated mice compared with the controls (Fig. 3A). Both, LTB_4_ and 5-HETE, are known to be chemoattractant to neutrophils promoting their migration to the site of inflammation [26], [27], [28]. In addition, in vitro 5-HETE has been shown to activate directly TRPV1 [29]. In irradiated rat skin 5- and 15-HETE were increased compared to the control tissue (Figure S1). In contrast to the results in the skin, in L4–L6-DRGs of both irradiated mice and rats only the concentrations of 12-HETE, an endogenous TRPV1 agonist [30], were increased in both tissues (Fig. 3B, Figure S1). In corresponding sections of the dorsal horn no changes in the levels of LOX-metabolites could be observed in mice or rats (Fig. 3C, Figure S1).

Similar to prostanoids and leukotrienes, LPAs with different chain lengths and saturation states 16∶0, 18∶0, 18∶1, 18∶2 were significantly elevated in the inflamed skin as compared to the control tissue (Figs. 3D). In contrast, LPA levels were not altered in lumbar DRGs and the dorsal horn (Figs. 3E, 3F). The oxidized linoleic metabolites 9- and 13-hydroxyoctadecadienoic acids (9- and 13-HODE) are endogenous TRPV1-agonists, which are elevated in heated rat skin [8], [31]. Both 9–and 13-HODE were elevated in the irradiated murine skin (Fig. 3G), while their levels did not change in the DRGs or spinal dorsal horn tissue as compared to the untreated mice (Figs. 3H, 3I). Taken together the data show so far that there is a strong upregulation of the synthesis of several lipid species in the skin, while there is, with the exception of 12-HETE, no increased lipid synthesis seen in DRGs or the spinal cord. These data suggest that lipid signaling is mainly involved in peripheral responses to UVB irradiation and seems not to play a major role in potential central mechanisms of UV-B-induced hyperalgesia.

**Figure 4:**
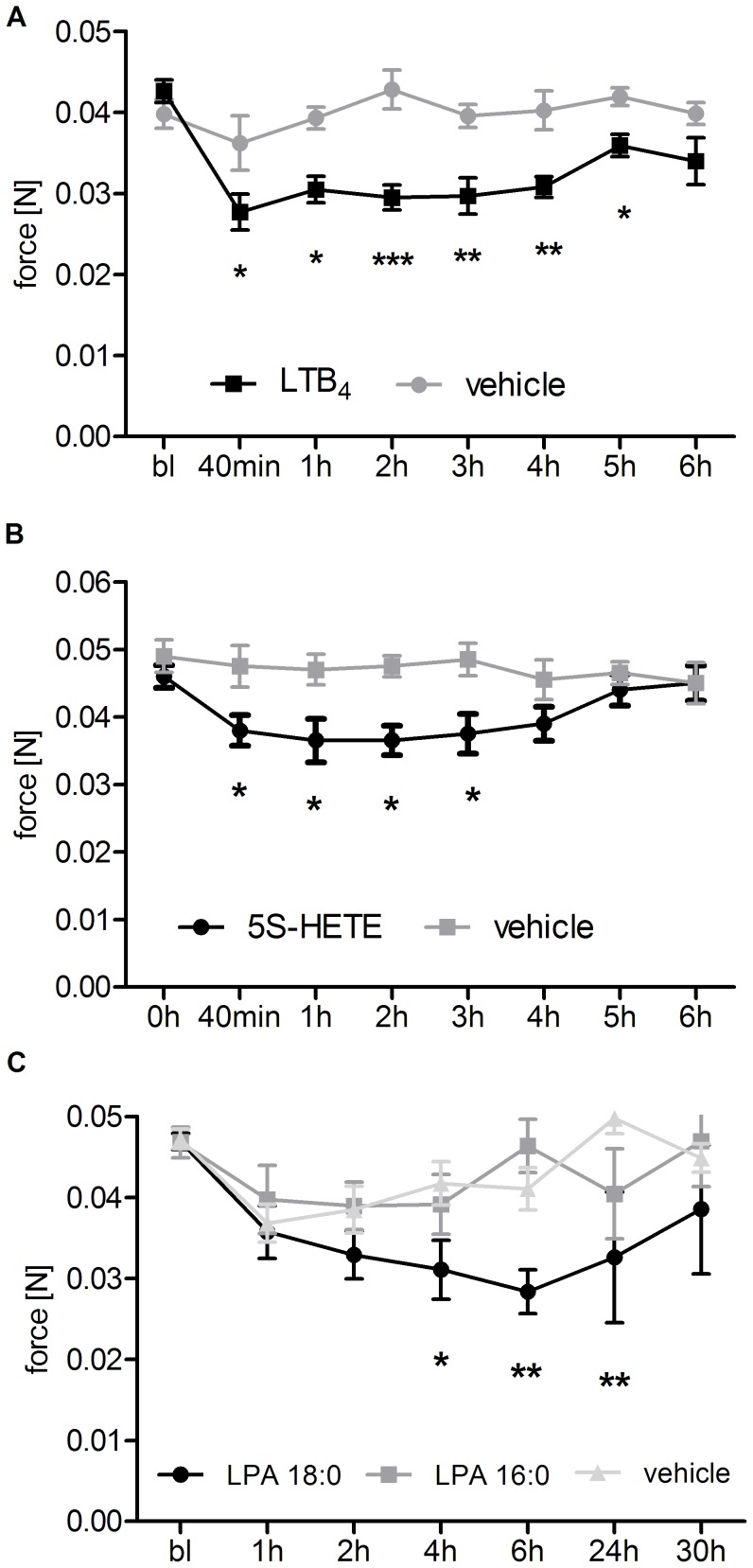
Mechanical thresholds of C57/Bl6 mice after injection of LTB4, 5-HETE, LPA 16∶0 or LPA 18∶0. Shown are paw withdrawal thresholds of wild type BL6 mice after intraplantar injection of LTB_4_ (10 µl of a 6 µM solution, A), 5-HETE (10 µl of a 6 µM solution B), LPA 16∶0 and LPA 18∶0 (both 10 µl of a 10 µM solution, C) and the corresponding vehicle (0.4% Ethanol (v/v) for LTB_4_ and 5-HETE, 1% DMSO (v/v) for LPA 16∶0 and LPA 18∶0). Mechanical thresholds were monitored until 6 h post injection (LTB4 and 5-HETE) or until 30 h post injection (LPA 16∶0 and LPA 18∶0). Data represent mean ± SEM from 6–8 mice per group (LTB_4_ and 5-HETE) or 7–11 mice per group (LPA 16∶0 and LPA 18∶0); *p<0.05, **p<0.01, ***p<0.001 two way repeated-measures ANOVA with Bonferroni post-hoc test. Structures were obtained from lipidmaps.org.

Next we addressed the question, whether or not the lipids, which were found to be increased in irradiated skin, can evoke mechanical allodynia. Since prostanoids, 9- and 13-HODE, as well as LPA 18∶1 have already been shown to cause mechanical allodynia upon intraplantar injection in mice [1], [8], [9], we injected LTB_4_, 5-HETE (both 10 µl of a 6 µM solution), LPA 16∶0 and LPA 18∶0 (both 10 µl of a 10 µM solution) in hind paws of mice and determined mechanical thresholds. Indeed, injection of LTB4 and 5-HETE caused a significant reduction of mechanical thresholds for 5–6 hours (Figs. 4A, 4B). Moreover, LPA 18∶0 but not LPA 16∶0 caused significant and long lasting reduction of mechanical thresholds four to 24 hours after intraplantar injection in mice (Figs. 4C). Thus, with the exception of LPA 16∶0, all lipids, which are upregulated in the skin after UVB irridation, are able to induce mechanical allodynia in mice. These data strongly suggest that the nociceptive response to UV-irradiation is based on several mediators, which origin from different COX-independent metabolic pathways.

**Figure 5:**
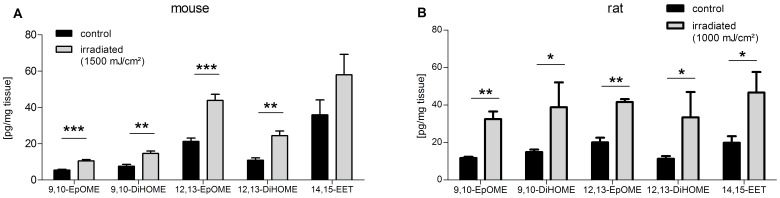
Comparison of epoxylipid-levels in skin tissue from irradiated mice and rats. Shown are the concentrations of 9,10- and 12,13-EpOME, and their metabolites 9,10–12,13-DiHOME, as well as 14,15-EET in skin from irradiated mice (A) and rats (B). Data represent mean ± SEM from five rats and six mice per group; *p<0.05, **p<0.01, ***p<0.001, student’s t-test.

The well described model of UVB induced skin inflammation [20], [21], [22] is thought to have a high translational potential [22]. However, since the number of genes for CYP enzymes differ strongly between mice on one side and rats and humans on the other side [23], we put a special focus on the comparison of levels of CYP-derived lipids. To compare lipid alterations in mice and rats, we irradiated rats as previously described with a UVB-dose of 1000 mJ/cm^2^ [16]. Both arachidonic acid and linoleic acid can be converted to epoxy-lipids by CYP-epoxygenases [32], [33], [34]. Members of the epoxyeicosatrienoic acids (EETs), epoxy-metabolites of arachidonic acid, are endogenous modulators of the transient receptor potential (TRP) ion channels TRPV4 and TRPA1 [35], [36]. Moreover, DiHOMEs, hydroxy-metabolites of linoleic acid, formerly called “leukotoxin-diols” have been shown to be produced in inflammatory leukocytes and display cytotoxic effects by causing respiratory burst [37], [38]. We determined the concentrations of EpOMEs (epoxy-metabolites of linoleic acid) and their dihydro-metabolites DiHOMEs, as well as EETs in skin samples of both irradiated rats and mice. Among the group of EETs, only 14,15-EET was detectable in these tissues. In irradiated murine skin samples all measured lipids from this group except 14,15-EET were significantly elevated (Fig. 5A). Similarly, in irradiated rat skin samples the concentrations of all detectable lipids from this group were significantly increased (Fig. 5B). In summary, although there is a profound difference in the number of CYP genes between rats and mice, the synthesis of lipids metabolized through CYP enzymes in response to UVB irradiation is very similar in both species.

## Discussion

Oxidized metabolites of arachidonic acid and linoleic acid as well as lysophosphatidic acid (LPA) 18∶1 have already been shown to activate TRP-channels in sensory neurons, leading to enhanced thermal or mechanical hyperalgesia during inflammation [8], [9], [30]. Here we combined a UVB-model of local skin inflammation and an analytical approach to investigate whether or not the concentrations of prostanoids, leukotrienes, lysophosphatidic acids and CYP-generated lipids are altered at the site of inflammation, in the DRGs and in the dorsal horn of the spinal cord. Interestingly, among all investigated lipid groups, the strongest concentration increases were observed at the site of inflammation (Table 1).

**Table 1:** Overview of lipid synthesis during UVB-induced inflammatory pain in skin, DRG and dorsal horn tissue from C57Bl6/N mice and Sprague Dawley rats.

	skin					DRGs					dorsal horn					
	Prost	HETE	LPA	CYP	HODE	Prost	HETE	LPAs	CYP	HODE	Prost	HETE		LPAs	CYP	HODE
mouse	↑	↑	↑	↑	↑	∼	↑ 12-HETE	∼	∼	∼	∼		∼	∼	↑14,15-EET	∼
rat	↑	↑	↑	↑	↑	∼	↑ 12-HETE	∼	∼	∼	↑PGD_2_		∼	∼	∼	∼

The tissue was dissected from six mice and six rats per group 48 hours post irradiation (1500 mJ/cm^2^ for mice and 1000 mJ/cm^2^ for rats) and lipids were extracted and quantified with LC-MS/MS. Prost: prostanoides, arrow indicates upregulation;

∼indicates no significant difference.

PGE_2_ is capable of sensitizing primary afferent neurons by binding one of its four G-protein coupled receptors (EP1-EP4) leading to TRPV1-sensitization through the PLC-PKC pathway (EP1) or the cAMP-PKA-pathway (EP2 and EP4) and contributing to thermal and mechanical hyperalgesia [39], [40]. In the group of measured prostanoids, both PGE_2_ and TXB_2_ were found to be elevated in irradiated skin tissue of mice, showing that cyclooxygenase-2 and the terminal PGE-synthases and TXA-synthase are upregulated during peripheral inflammation. Additionally, PGF2α was found to be elevated after irradiation, indicating that PGI-synthase is specifically activated in mice during peripheral UVB-induced skin inflammation. Moreover, the concentrations of LPA 18∶1, 9-HODE were significantly elevated in the inflamed skin tissue of mice. These lipids have been reported to be either direct or indirect activators of TRPV1 [8], [9] and may thus contribute to thermal hyperalgesia and mechanical allodynia during UVB-induced inflammation. Fittingly, 9-HODE has been shown to be generated in heated skin and recently HODEs were shown to be markedly upregulated in murine skin biopsies in a post-burn pain model of partial-thickness Injury [8], [41].

Moreover, we show that LTB4, 5-HETE, LPA 16∶0 and LPA 18∶0 increased in inflamed skin tissue. LTB_4_, and 5-HETE caused a significant reduction of the mechanical thresholds lasting up to six hours post injection. Additionally, injection of LPA 18∶0 but not LPA 16∶0 resulted in long lasting decreased mechanical thresholds four to 24 hours after injection, indicating an indirect and possibly secondary sensitizing function for LPA 18∶0 in peripheral inflammatory hyperalgesia. Several explanations for the pronociceptive effects of the 5-LO products 5S-HETE and LTB4 have been described that may explain their effects on pain thresholds. For example, LTB4 receptors which are expressed on peripheral sensory neurons are thought to be involved in the sensitization of nociceptors [42], while 5S-HETE can directly activate TRPV1 [43]. Also, both lipids are able to activate cytosolic phospholipase A_2_ (PLA_2_) and, therefore, to stimulate the synthesis of pronociceptive prostaglandins (i.e. PGE_2_) [44]. Finally, LTB4 and 5S-HETE have a strong chemoattractive potential causing neutrophil recruitment [27], [28] or monocyte migration [45].

These results may explain, why COX-inhibitors administered even at high doses have relatively weak antinociceptive effects in reversing thermal hyperalgesia or mechanical allodynia in irradiated rats [16], [17], [18], [19]. The upregulation of COX-independent TRPV1 agonists LPA 18∶1, 9-HODE and 5-HETE and other proalgesic acting lipids, such as LTB_4_ and LPA 18∶0 may still cause activation and/or sensitization of TRPV1 and subsequently thermal hyperalgesia and mechanical allodynia even if prostanoid synthesis is inhibited. According to these results, a selective TRPV1-antagonist may be more effective for treating UVB-induced inflammatory pain than cyclooxygenase inhibitors.

Epoxylipids are generated by CYP-epoxygenases of the subfamilies 2C and 2J [34]. Interestingly almost all measured epoxylipids and metabolites were found to be elevated in irradiated skin of both rats and mice, leading to the conclusion that upregulation of CYP epoxygenases 2C and 2J and possibly of phospholipase A_2_, delivering arachidonic acid and linoleic acid as substrate, occurs in both species during UVB-induced skin inflammation. Notably, mice and rats are equipped with a different number and isoform-constellation of CYP-epoxygenases [23]. However, given the concentration differences of epoxylipids in irradiated skin of both species, these different isoforms do not differ in generation or preference of epoxylipids between the two investigated species, and seem to be regulated similarly during peripheral inflammation. This is consistent with previous findings investigating the synthesis and regulation of epoxylipids in rats and mice under pathophysiological conditions in the cardiovascular context [46]. Ruparel et al. showed upregulation of CYP2J4 in trigeminal ganglia (TGs) of rats during CFA-induced inflammatory pain, and showed CYP2J4 expression in TG-neurons, thus pointing toward a role of CYPs and CYP-derived lipids in inflammatory pain [47]. We also found CYP-lipids in increased concentrations during UVB-induced inflammatory pain, however not in DRGs, but in the dorsal horn and most predominantly in the skin. These different regulatory locations of CYP-epoxygenases may be due to differences of the inflammatory models. It is still unclear which cells produce CYP-derived lipids in the skin, but it is possible that they are released by resident immune cells as part of oxidative stress response during UVB-induced skin inflammation.

Recently, TRPV4 expressed in keratinocytes has been exposed to be involved in generating UV-dependent inflammatory hyperalgesia. However, the involvement of endogenous TRPV4 modulators has not been investigated [48]. Perhaps synthesis of oxidized lipids as endogenous TRPV4 activators and the subsequent neuronal and immune cell responses is a necessary regulatory step in generating UVB-dependent inflammatory pain.

It is unclear why the observed concentration changes are most exclusively located in the periphery and at the site of inflammation. These findings are consistent with the observation that hyperalgesia following UVB is thought to principally be mediated by peripheral sensitization [20].

One may speculate that the area of skin inflammation is small and that only a part of the nociceptors in the skin are activated, leading to comparably low nociceptive input to the dorsal horn as compared to an invasive model of inflammation such as carrageenan or complete freud’s adjuvant (CFA) This also leads to minor changes in lipid concentrations in the central nervous system.

In summary, members of LPAs, Epoxylipids, HODEs, leukotrienes and prostaglandins were found to be significantly increased in skin samples from mice in a UVB-model of peripheral inflammation. In particular, we found already known endogenous TRPV1-agonists, such as HODEs, LPA 18∶1, 5- and 12-HETE to be increased in irradiated skin. Moreover, LPA 18∶0, a lipid that was not formerly related to inflammatory pain, was found in increased concentrations in irradiated skin and caused long lasting mechanical allodynia in mice when injected intraplantarly. The high abundance of these proalgesic COX-independently generated lipids may explain, why COX-inhibitors such as ibuprofen only show weak antinociceptive effects in UVB-induced mechanical allodynia in rodents [16], [17], [18], [19] and indicate that TRPV1-antagonists may be more promising in treating UVB-induced inflammatory pain.

## Supporting Information

Figure S1 **Prostanoid- and HETE-levels in skin, DRG and spinal dosal horn tissue from irradiated SD-rats.** Concentrations of prostanoids from isolated skin (A) L4–L6-DRGs (B) and the corresponding section of the spinal dorsal horn (C) from irradiated (1000 mJ/cm^2^, grey bars) versus untreated rats (black bars). (D–F) Levels of HETEs in skin (D), DRG (E) and dorsal horn tissue (F) from rats. Data represent mean ± SEM from five rats per group; *p<0.05, **p<0.01, student’s t-test, n.d, not detectable.
(TIF)
