# Supplementary figures and images for: Synthesis of Lipid Mediators during UVB-Induced Inflammatory Hyperalgesia in Rats and Mice
Source: PLoS One. 2013 Dec 9;8(12):e81228. doi: 10.1371/journal.pone.0081228 (PMC3857181; doi:10.1371/journal.pone.0081228)

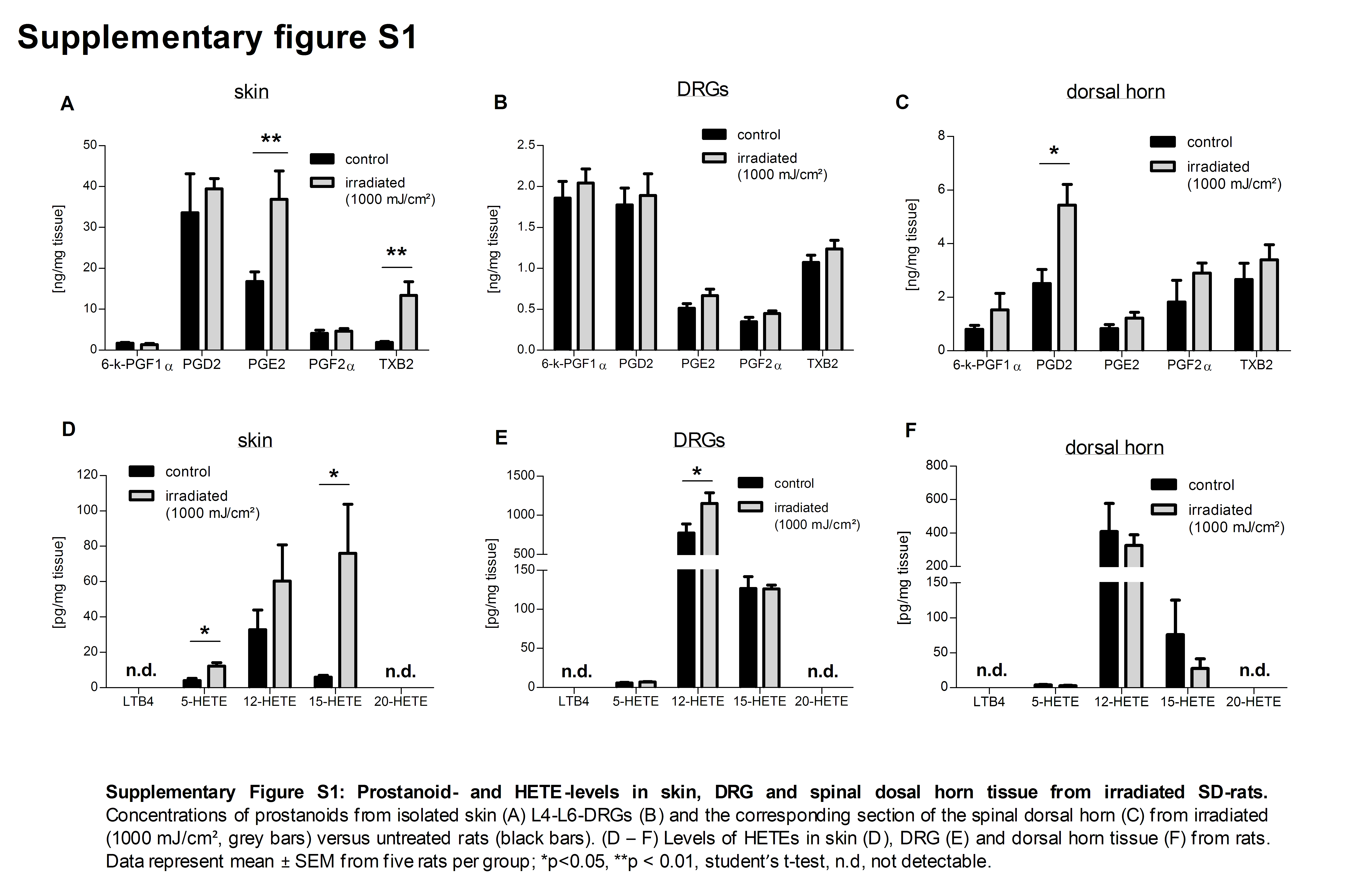

Supplement: Figure S1 — Prostanoid- and HETE-levels in skin, DRG and spinal dosal horn tissue from irradiated SD-rats. Concentrations of prostanoids from isolated skin (A) L4–L6-DRGs (B) and the corresponding section of the spinal dorsal horn (C) from irradiated (1000 mJ/cm2, grey bars) versus untreated rats (black bars). (D–F) Levels of HETEs in skin (D), DRG (E) and dorsal horn tissue (F) from rats. Data represent mean ± SEM from five rats per group; *p<0.05, **p<0.01, student’s t-test, n.d, not detectable. (TIF) [file pone.0081228.s001.tif]
